# Syndemic Synergy of HPV, HIV, and HSV-2 for Oncogenic HPV Replication in Female Sex Workers

**DOI:** 10.3390/tropicalmed10060157

**Published:** 2025-06-07

**Authors:** Jonathan Muwonga Tukisadila, Ralph-Sydney Mboumba Bouassa, Serge Tonen-Wolyec, Hugues Loemba, Jeremie Muwonga, Laurent Belec

**Affiliations:** 1 École Doctorale Régionale D’Afrique Centrale en Infectiologie Tropicale, Franceville 876, Gabon; ralphsmbouassa@montfort.on.ca (R.-S.M.B.); wolyec@gmail.com (S.T.-W.); 2 Laboratoire de Biologie Clinique des Cliniques Universitaires de Kinshasa, Kinshasa 123, Democratic Republic of the Congo; pmuwonga@hotmail.com; 3 Laboratory of Virology, Hôpital Européen Georges Pompidou, Assistance Publique-Hôpitaux de Paris (AP-HP), 75015 Paris, France; laurent.belec@aphp.fr; 4 Institut du Savoir Montfort, Montfort Hospital, Ottawa, ON K1K 0T2, Canada; huguesloemba@montfort.on.ca; 5 Department of Family Medicine, Faculty of Medicine, University of Ottawa, Ottawa, ON K1N 6S1, Canada; 6 Department of Internal Medicine, Faculté de Médecine, Université de Bunia, Bunia 292, Democratic Republic of the Congo; 7 Faculté de Médecine Paris Descartes, Université Paris Cité, 75006 Paris, France

**Keywords:** cervical cancer, human papillomavirus (HPV), self-sampling, genital veil-based collector device, HPV detection and genotyping, HSV-2, multiplex real-time PCR, HPV viral load, female sex workers, sub-Saharan Africa

## Abstract

Background: Female sex workers (FSWs) in sub-Saharan Africa bear a disproportionate burden of sexually transmitted infections, including HIV, high-risk HPV (HR-HPV), and herpes simplex virus type 2 (HSV-2). This study evaluated possible association between HR-HPV, HIV, and HSV-2 among FSWs in the Democratic Republic of the Congo. Methods: A cross-sectional study was conducted among 432 FSWs (mean age, 28.1 years) recruited via respondent-driven sampling. Genital self-sampling using the V-Veil UP2™ device was performed, followed by HPV genotyping and quantification by multiplex PCR, and HSV-2 DNA detection by PCR. Results: Among 415 participants, HR-HPV prevalence was 36.9%, with HPV-52 (14.9%), HPV-58 (10.1%), and HPV-16 (6.5%) as leading genotypes. Overall, 89% of HR-HPV-positive women harbored genotypes covered by Gardasil-9^®^. Co-infection with HIV and HSV-2 significantly increased HPV prevalence, genotype diversity, and viral load. Notably, HSV-2 positivity was the sole independent predictor of elevated replication of HR-HPV (*p* < 0.001), vaccine HR-HPV (*p* < 0.001), and non-vaccine HR-HPV (*p* < 0.021). Conclusions: FSWs exhibit a high burden of HR-HPV, shaped by co-infections with HIV and HSV-2. HSV-2 independently drives HR-HPV replication, highlighting its role in HPV persistence and cervical cancer risk. Integrated HSV-2 detection and Gardasil-9^®^ vaccination should be prioritized in cervical cancer elimination strategies targeting high-risk populations in sub-Saharan Africa.

## 1. Introduction

Female sex workers (FSWs) in sub-Saharan Africa (SSA) are disproportionately affected by a complex interplay of individual, network, and structural risk factors that heighten their vulnerability to poor health outcomes. An estimated 2.5 million women aged 15–49 engage in sex work across SSA, comprising approximately 1.1% (95% Uncertainty Interval: 0.8–1.3%) of the adult female population [1]. FSWs in SSA represent a key population in the burden of sexually transmitted infections (STIs), including human immunodeficiency virus (HIV), high-risk human papillomavirus (HR-HPV) and associated cancer, and herpes simplex virus type 2 (HSV-2). Indeed, HIV incidence and prevalence remain disproportionately elevated among FSWs compared to the general population [2,3,4,5,6]. Recent estimates suggest an eightfold higher HIV incidence among FSWs, despite a 67% decline in new infections over the past decade [5,7]. Accordingly, the HIV epidemic remains concentrated among FSWs, who continue to serve as a critical reservoir for the generalized epidemic in SSA [3,7,8].

FSWs are also disproportionately affected by HR-HPV and associated cancers. Cervical cancer remains the second leading cause of cancer-related mortality among women in SSA [9,10,11,12], and FSWs exhibit increased prevalence of HR-HPV genotypes and higher incidence of precancerous Cervical Intra-epithelial Neoplasia (CIN) compared to the general population [13,14,15,16]. This elevated risk is compounded by multiple cofactors, including early sexual debut, multiple sexual partners, poverty, social stigmatization, tobacco use, low educational level, and limited access to healthcare and screening, contributing to delayed diagnosis and poor outcomes [14,16,17].

Similarly, FSWs in SSA have an increased risk of genital HSV-2 with both incidence and seroprevalence estimates exceeding those in the general adult female population [18,19,20,21]. The elevated burden of HSV-2 in FSWs is of great concern as HSV-2 contributes to mucosal disruption and local immune modulation, potentially facilitating acquisition and persistence of HIV and HR-HPV [22,23,24,25,26].

Finally, the FSWs in Africa represent a very particular core group of several STIs such as HIV, HPV, and HSV-2 infections, which could have syndemic synergy for HPV-associated cervical lesions, where disease/outcome risk is seen as a combination of biological, behavioral, and social factors [27]. Indeed, HSV-2 infection increases the risk of HIV acquisition and transmission in sub-Saharan Africa [28,29], and HIV infection promotes HSV-2 replication and HIV disease progression [30]. Otherwise, HIV-positive women are more at risk for progression to CIN grade 3 [13,31,32,33]. Furthermore, co-infections with HSV-2 serve as an independent predictor for HPV genital shedding [34,35] and for precancerous and cervical cancer among sexually active community [25,36,37].

The Democratic Republic of the Congo (DRC) is the fourth most populous country in Africa, as well as the most populous French-speaking country, with 111 million inhabitants. The HIV epidemics remains generalized [adult prevalence: 1.1%, 95% confidence interval (CI): 1.0–1.4%] and while FSWs account for nearly 1% of the total adult population, the HIV burden among FSWs is over six times higher (7.5%) [7]. Cervical cancer is the most common and deadliest cancer among Congolese women, and HR-HPV prevalence exceeds 25% in Kinshasa [38,39]. Moreover, HSV-2 seroprevalence among FSWs in Kinshasa approaches 60% [18].

Given this syndemic burden, this study investigates the prevalence of HPV genotypes, HIV, and genital HSV-2 shedding among FSWs living in Kisangani, DRC. We further assess the associations between these infections and relevant demographic and behavioral factors and explore the interplay between type-specific HPV viral load, HIV infection, and HSV-2 co-infection.

## 2. Materials and Methods

### 2.1. Study Design, Population, and Recruitment

This multicenter, population-based, cross-sectional study was conducted in 2022 among FSWs recruited from the community in Kisangani, the capital of the Tshopo province in the DRC. Data were collected using structured face-to-face questionnaires following the Standards for Reporting of Diagnostic Accuracy (STARD) guidelines [40]. The study aimed to assess the prevalence and association of genital HPV, including HR-HPV, possibly oncogenic HPV (PO-HPV), and low-risk HPV (LR-HPV) types, as well as HIV and HSV-2. FSWs were defined as women aged 18–65 who reported more than two sexual partners (excluding their regular partner) in the prior three months and received money or “gifts” in return for sexual activity [20,41]. “Professional FSWs” self-reported sex work as their main income source and were categorized based on work setting (bars/hotels versus poor neighborhoods). “Non-professional FSWs” engaged in transactional sex but identified another main source of income or were students. These were subdivided into two categories, including “occasional” (e.g., schoolgirls or students) and “regular” (e.g., street vendors, housewives, unemployed women, civil servants).

Inclusion criteria included: being an FSW, aged 18–65, ability to understand HIV self-test instructions, willingness to undergo HIV testing and receive the results, and informed written consent. Exclusion criteria included: male biological sex, age outside eligibility rate, dry sex practice in the prior 48 h, symptomatic STIs, plastic or cotton allergies, or refusal to comply with the protocol. None of the participants had received HPV vaccination or were under antiretroviral or anti-herpetic treatment at the time of inclusion.

The so-called “Progrès Santé Sans Prix“ is a specialized non-governmental organization (NGO) exclusively dedicated to key populations in the DRC. It offers counseling, testing, care, and support to key populations, including FSWs. In the Kisangani province, FSWs regularly attend the NGO center established in the Makiso suburb, which is devoted to HIV and STIs screening and care, in order to receive specific treatment, HIV counseling, and HIV global support for those tested positive. All participants volunteered to take part in the study.

The study used the respondent-driven sampling (RDS) method to deliberately select eight initial FSW participants (referred as “seeds”) that were purposively selected by the investigators as they were well-connected with FSW networks in the Kisangani province, to serve as initial contacts for chain recruitment of FSWs through their network, as previously described [42]. The five suburbs of the Kisangani province, including Kisangani, Makiso, Tshopo, Kabondo, and Mangobo suburbs, were involved in the study. All initial seeds were given five coupons to recruit FSWs from among their peers. Recruited FSWs became secondary seeds who were given four coupons. Finally, new eligible and consenting seeds, became themselves recruiters. Recruits were encouraged to take part in the study, without being paid, and to recruit other FSWs of their network. This process was carried out in several waves of recruitment (with wave zero being defined as the seeds, wave one as their recruits, etc.). Due to the absence of any previous knowledge on the prevalence of genital HPV infections among FSWs in the DRC, no pre-determined sample size was fixed, and the recruitment was carried out by RDS until the network was exhausted. The promotion and visibility of this survey were conducted by sensitizing people at the study hot spots by distributing visiting tokens to anyone during the day as well as night.

In each inclusion suburb, the seeds contacted adult FSWs in hot spots, including dance-halls, bars, hotels, and brothels, during a two-month period and proposed them to be included in the study after an oral explanation on the objectives of the survey, mainly focused on sensitization on cervical cancer and prevention strategies against cervical HPV acquisition and HIV infection. The selected FSWs were invited, with paid transportation, to come to health centers in the Makiso suburb. The choice of these health facilities was justified by their integrating of HIV prevention and care packages, including STIs, providing free care for people living with HIV, and easier accessibility for persons sensitized at the hot spots.

### 2.2. Study Procedure and Sample Collection

The participants were looked after by trained research assistants (physicians or nurses) in groups of ten, and given advice on how to use the genital device for vaginal self-testing and HIV self-testing, and on cervical cancer diagnosis and prevention. After explaining the study’s objective to the participants, written informed consent was obtained. Then, the participants were asked to respond to a self-administrated survey questionnaire regarding their age, socio-demographic characteristics, number of sexual partners within the last 3 days, and HIV testing history, and received adequate pre-test HIV counselling. The study participant received from a nurse a 15-min training on how to use the Vaginal Veil Collector V-Veil UP2™ (V-Veil-Up Production SRL, Calinesti, Romania; https://hpv-veil.com), which is listed by UNITAID [43]. Furthermore, participants were given leaflets with pictures illustrating the procedure for HIV self-testing and self-collection. After instructing the participant, the nurse left the sampling room allowing privacy and the participant then performed herself the self-test and the self-sampling, without any help from the nurse. The participant followed the instructions for use of the vaginal veil, which was let in place during 30 min. The nurse checked that the sample had been taken correctly, that the tube had been closed properly, and that the identification on the label matched. HIV testing

HIV serostatus was determined using the CE IVD Exacto HIV^®^ self-test (Biosynex, Strasbourg, France), previously validated for field use in the DRC with 100% sensitivity and 98.9% specificity [44,45,46]. Participants with reactive results were referred to confirmatory testing as per the national algorithm [47]. Confirmed HIV-positive individuals were linked to care services, and post-test counseling was provided. HIV-negative participants received counseling on risk reduction strategies and, if interested, pre-exposure prophylaxis (PrEP).

### 2.3. Genital HPV Sampling and Transport

Vaginal specimens collected via self-sampling were placed into 50 mL conical tubes and stored at −30 °C [48]. Frozen impregnated veils were transported on dry ice to the virology laboratory at Hôpital européen Georges Pompidou, Paris, France. For processing, veils were placed in 10 mL of Cyt-All^®^ universal transport medium (Alphapath, Mauguio, France; https://www.alphapath.fr/cyt-all/ accessed on 5 May 2025).

### 2.4. DNA Extraction, HPV Genotyping, and Quantitation by Multiplex PCR

DNA was extracted from 200 µL of cervicovaginal secretions using the QIAamp^®^ DNA Mini Kit (Qiagen, Hilden, Germany), eluted in 100 µL of elution buffer, and stored at −30 °C. HPV genotyping was performed using the Bioperfectus Multiplex Real Time PCR kit (BMRT HPV genotyping Kit, Jiangsu Bioperfectus Technologies Co., Ltd., Taizhou, Jiangsu Province, China), which targets a 100-bp fragment of the L1 gene as well as an internal control with the housekeeping single-copy gene TOP3 encoding human DNA topoisomerase III in reaction tube H (FAM™ channel) to identify possible PCR inhibition and to confirm the reliability of the reagents in this kit as well as the viral loads simultaneously [49]. This assay detects 21 genotypes: 13 HR-HPVs (HPV-16, -18, -31, -33, -35, -39, -45, -51, -52, -56, -58, -59, and -68), five PO-HPVs (HPV-26, -53, -66, -73, and -82) and three LR-HPVs (HPV-6, -11, and -81) [50]. The BMRT HPV Genotyping Real Time PCR assay allowed to determine the normalized HPV viral load of the 21 detected HPVs, expressed as log (normalized viral copies per 10,000 human cells), by integrating the cycle threshold (Ct) values of the emitted fluorescence of L1 gene of each HPV and the Ct of the sample TOP3 gene, using the Bioperfectus HPV Analyzer Software v1.0 (Jiangsu Bioperfectus Technologies Co., Ltd., Taizhou, China), as described [49].

### 2.5. HSV-2 DNA and Prostatic-Specific Antigen (PSA) Detection

HSV-2 DNA was detected using qualitative PCR following previously validated protocols [51]. Prostatic-specific antigen (PSA) was used as a biomarker of recent semen exposure, measured by chemiluminescent microparticle immunoassay [52,53].

### 2.6. Statistical Analysis

Data were entered into Excel and analyzed using standard statistical software. Quantitative variables were summarized using means and standard deviations (SDs), and categorical variables as proportions with 95% CI, calculated using Wilson’s method [54]. Prevalence was determined for overall HPV infection, as well as by HR-HPV, PO-HPV, LR-HPV, and 4- and 9-valent Gardasil^®^ vaccine types (Merck & Co. Inc., Rahway, NJ, USA). HPV viral loads in genital specimens as assessed by BMRT HPV Genotyping Real Time PCR assay were expressed as copies per 10,000 cells, and log-transformed. In case of coinfections, the HPV viral load used in the evaluation was the cumulative HPV loads in the genital specimen. HPV viral load was log-transformed and evaluated cumulatively in the case of co-infections. Association between categorical variables were tested using Pearson’s χ^2^ or Fisher’s exact test, while continuous variables were analyzed using the Mann–Whitney U-test or Kruskal–Wallis rank sum test, using the BiostatTGV facilities available online (https://biostatgv.sentiweb.fr/). A two-tailed *p*-value < 0.05 was considered statistically significant. To identify predictors of HR-HPV replication, multiple linear regression models were built using DATAtab software version 1 (DATAtab e.U. Graz, Austria; https://datatab.net). Independent variables included the 10 sociodemographic and behavioral factors, and the infectious profile of the participants (2 variables: HIV infection and HSV-2 DNA positivity). The primary dependent variable was the “HR-HPV total viral load,” which represented the cumulative viral load of all HR-HPV genotypes detected in a single genital sample, expressed as log copies/10,000 cells. Additional dependent variables included the “Gardasil-9^®^ HR-HPV total viral load” and “non-vaccine HR-HPV total viral load,” corresponding to the viral load of HR-HPV genotypes targeted by the Gardasil-9^®^ vaccine and those not covered by the vaccine, respectively. For each dependent variable, the regression coefficient (β) of each independent variable was calculated, along with its 95% CI. A positive β indicates a positive association with the dependent variable, while a negative β indicates a negative association. An effect was considered statistically significant if the β coefficient differed from zero, with the 95% CI excluding zero, and the *p*-value was less than 0.05.

### 2.7. Ethical Considerations

The study is research with direct individual benefit, including the virological diagnosis of oncogenic HR-HPV. Written informed consent was obtained by each participant after explaining the purpose of the study. The work was conducted in accordance with the Ethical Guidelines of the World Medical Association Declaration of Helsinki. The study was approved by the Institutional Review Board of institutional ethical committee at the University of Kisangani, Kisangani, DRC, and at the Ecole Doctorale en Infectiologie Tropicale of the Masuku University, Franceville, Gabon.

## 3. Results

### 3.1. Study Population

A total of 432 participants were enrolled. Of these, only 17 declined self-collection, yielding a high acceptability rate for the V-Veil UP2™ device (96.1%, 415/432). Table 1 presents the sociodemographic and behavioral characteristics of the 415 who completed self-sampling.

The mean age was 28.2 years, with even distribution across the five suburbs of Kisangani. Christianity was the predominant religion (55.7%). Most participants had a low educational level (61.1%), while 31.1% completed secondary school and 7.8% held university degrees. The majority were unmarried (80.2%). Nearly half (47.9%) identified as professional FSWs, most working in poor neighborhoods (33.0%), and others in bars or hotels (14.9%). Among non-professional FSWs, the regular ones (e.g., vendors, employed) made up 47.0%, and the occasional (schoolgirls/students), 5.1%. Daily income was typically low, with 43.1% earning under USD 5. All FSWs were sexually active, with at least one male partner in the past three days; 10% reported more than five partners during that time. PSA testing confirmed recent semen exposure in one-third of FSWs, indicating unprotected intercourse.

Previous HIV testing had been performed by 57.1% of participants. The overall HIV prevalence was 9.1% (95%CI: 6.3–11.9%). High HIV seropositivity was significantly associated with age > 40 years, residence in Makiso or Kabondo, the Kimbanguist religion, primary education level, professional FSW status, higher income (>USD 10 daily), and multiple partners (>5 in past 3 days). Conversely, HIV-negative status was linked with living in Tshopo or Mangobo, Christian affiliation, non-professional FSW status, mid-range daily income, fewer recent partners, and previous HIV testing.

Genital HSV-2 DNA was detected in 24.3% (95%CI: 20.3–28.5%) of participants and was 3.3 times more prevalent in HIV-infected FSWs (*p* < 0.0001).

### 3.2. HPV Prevalence and Genotypes Distribution

**Figure 1:**
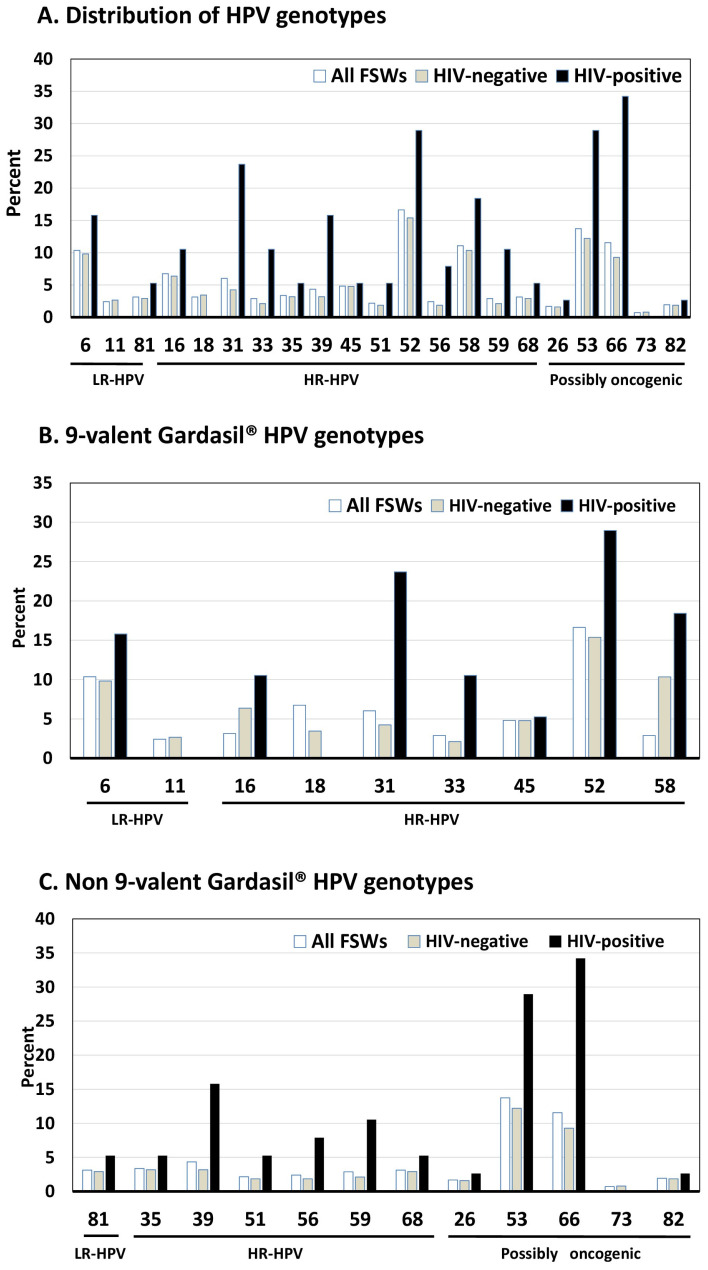
Distribution of HPV genotypes (in percentage) by HIV serostatus (negative or positive) among the 415 study adult female sex workers living in Kisangani, Democratic Republic of the Congo. (**A**) Low-risk HPV (LR-HPV), high-risk HPV (HR-HPV), and possibly oncogenic HPV (PO-HPV) genotypes; (**B**) LR-HPV and HR-HPV genotypes targeted by the 9-valent Gardasil-9^®^ vaccine; (**C**) LR-HPV, HR-HPV, and PO-HPV genotypes not targeted by the Gardasil-9^®^ vaccine. HPV genotypes were determined from veil-based collected genital samples positive for HPV molecular detection using the BMRT HPV Genotyping Real Time PCR assay (Bioperfectus Technologies Co., Ltd., Taizhou, Jiangsu, China). Nota bene. According to the manufacturer’s instructions and in accordance with the HPV classification nomenclature provided by the International Agency for Research on Cancer [50], the BMRT HPV Genotyping Real Time PCR Kit distinguishes 21 HPV genotypes, including 13 HR-HPV genotypes (HPV-16, -18, -31, -33, -35, -39, -45, -51, -52, -56, -58, -59, and -68), three LR-HPV types (HPV-6, -11, and -81) and four genotypes classified as possibly oncogenic (HPV-26, -53, -66, -73, and -82). Nota bene. The 9-valent Gardasil-9^®^ vaccine (Merck & Co. Inc., NJ, USA) targets the seven HR-HPV genotypes predominantly isolated in cervical cancer (HPV-16, -18, -31, -33, -45, -52, and -58) and two LR-HPVs (HPV-6 and HPV-11).

**Figure 2:**
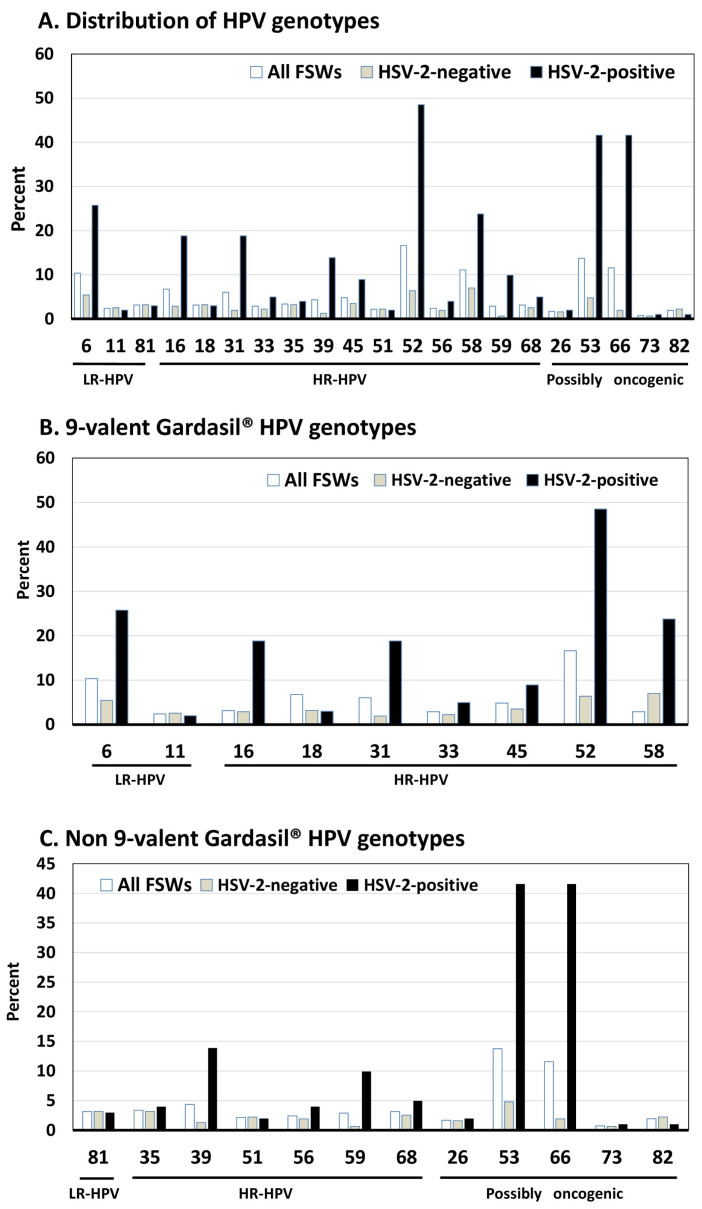
Distribution of HPV genotypes (in percentage) by genital HSV-2 DNA status (negative or positive) among the 415 study adult female sex workers living in Kisangani, Democratic Republic of the Congo. (**A**) Low-risk HPV (LR-HPV), high-risk HPV (HR-HPV), and possibly oncogenic HPV (PO-HPV) genotypes; (**B**) LR-HPV and HR-HPV genotypes targeted by the 9-valent Gardasil-9^®^ vaccine; (**C**) LR-HPV, HR-HPV, and PO-HPV genotypes not targeted by the Gardasil-9^®^ vaccine. HPV genotypes were determined from veil-based collected genital samples positive for HPV molecular detection using the BMRT HPV Genotyping Real Time PCR assay (Bioperfectus Technologies Co., Ltd., Taizhou, Jiangsu, China). Nota bene. According to the manufacturer’s instructions and in accordance with the HPV classification nomenclature provided by the International Agency for Research on Cancer [50], the BMRT HPV Genotyping Real Time PCR Kit distinguishes 21 HPV genotypes, including 13 HR-HPV genotypes (HPV-16, -18, -31, -33, -35, -39, -45, -51, -52, -56, -58, -59, and -68), three LR-HPV types (HPV-6, -11, and -81) and four genotypes classified as possibly oncogenic (HPV-26, -53, -66, -73, and -82). Nota bene. The 9-valent Gardasil-9^®^ vaccine (Merck & Co. Inc., NJ, USA) targets the seven HR-HPV genotypes predominantly isolated in cervical cancer (HPV-16, -18, -31, -33, -45, -52, and -58) and two LR-HPV (HPV-6 and HPV-11).

All veil specimens tested positive for the ubiquitous TOP3 gene, confirming successful sample collection. The overall prevalence of any HPV was 47.5% (95%CI: 42.7–52.3%), significantly higher in HIV-positive and HSV-2-positive participants (*p* < 0.0001). LR-HPV was found in 15.9% (95%CI: 12.4–19.4%), with HPV-6 being the most common, detected in 10.4% overall and 25.7% of HSV-2-positive specimens (*p* < 0.0001) (Table 2 and Figure 1 and Figure 2).

HR-HPV prevalence was 36.9% (95%CI: 32.3–41.5%), significantly higher in HIV-positive (*p* < 0.0005) and HSV-2-positive (*p* < 0.0001) women. While HPV-16 (6.7%), HPV-18 (3.1%), and HPV-45 (4.8%) were relatively infrequent, they were substantially more common among HSV-2-positive participants (HPV-16: 18.8%, HPV-45: 8.9%, *p* < 0.05).

Genotypes HPV-31, HPV-33, HPV-39, HPV-56, HPV-52, and HPV-59 were more detected in HIV-positive women. HSV-2-positive women showed significantly higher prevalence of HPV-16, HPV-31, HPV-39, HPV-45, HPV-52, HPV-58, and HPV-59.

PO-HPV was found in 26.9% (95%CI: 22.6–31.2%), with HPV-53 and HPV-66 as the most common types. Both were especially prevalent in HSV-2-positive participants (41.6%; *p* < 0.0001). Multiple infections were significantly more common in HIV-positive and HSV-2-positive women. Multiple HR-HPV infections (mean ± SD: 1.9 ± 1.5, range: 2–8 types), were found in 34.2% of HIV-positive and 43.6% of HSV-2-positive FSWs (*p* < 0.003 and <0.001, respectively). PO-HPV co-infections were also more frequent in HSV-2-positive FSWs.

Gardasil-4^®^ vaccine-HPV type prevalence was low. In contrast, Gardasil-9^®^-covered types were found in 39.5% of all participants, but significantly more in HIV-positive (63.2%) and HSV-2-positive (90.1%) women. The most common HR-HPV types covered by Gardasil-9^®^ were HPV-52 (16.6%), HPV-58 (11.1%), and HPV-16 (6.7%), followed by HPV-31 (6.0%), HPV-45 (4.8%), HPV-18 (3.1%), and HPV-33 (2.9%). LR-HPV types HPV-6 (10.4%) and HPV-11 (2.4%) were also frequent.

Notably, 89% (136/153) of HR-HPV-positive participants had at least one Gardasil-9^®^ HR-HPV genotype. However, 13.0% shed only non-vaccine HR-HPV types, including HPV-39 (4.3%), HPV-35 (3.4%), HPV-68 (3.1%), HPV-59 (2.9%), HPV-56 (2.4%), and HPV-51 (2.1%) (Table 2 and Figure 1).

### 3.3. Quantitative HPV Viral Load by HIV and HSV-2 Status

Table 3 shows the HPV viral load by according to HIV and HSV-2 status. LR-HPV viral load, particularly HPV-6, was significantly higher in HSV-2-positive FSWs. Among HR-HPV types, viral loads of HPV-31, HPV-33, HPV-39, HPV-52, and HPV-59 were higher in HIV-positive FSWs. HSV-2-positive women had significantly higher viral loads of HPV-16, HV-31, HPV-39, HPV-45, HPV-52, HPV-58, and HPV-59.

Cumulative viral loads for all HPV categories, including HR-HPV, PO-HPV, Gardasil-9^®^ HR-HPV, and non-vaccine HR-HPV types, were significantly elevated in HIV-positive and HSV-2-positive FSWs.

### 3.4. Factors of HR-HPV Replication Using Multiple Linear Regression Analysis

Multiple linear regression analyses identify HSV-2 infection as the unique independent factor significantly associated with increased HR-HPV replication. HSV-2 positivity was associated with elevated viral loads across all HPV categories, including HR-HPV (β: 5.32, *p* < 0.001), Gardasil-9^®^ HR-HPV (β: 4.59, *p* < 0.001), and non-vaccine HR-HPV (β: 0.84, *p* = 0.021). The variance inflation factor (VIF) for HSV-2 across all three models was low (VIF = 1.642), indicating no concerning multicollinearity with the other independent variables. No other socio-demographic or behavioral factors were significantly associated with HR-HPV replication (Table 4).

### 3.5. Summary of the Main Results

The main results of this study can be summarized as follows.

✓ The seroprevalence of HIV in the study FSWs reached 9.1%, around 8.3-fold higher than the general adult female population in the DRC, reinforcing that FSWs are a key population for HIV in a country of generalized HIV epidemic;
✓ The novel Vaginal Veil Collector V-Veil UP2™ device especially conceived for female genital self-sampling demonstrated high acceptability (~96%) and effectiveness for molecular biology (100.0%);
✓ The distribution of HPV in genital samples appeared atypical and unique, with a mixture of HR-HPVs, PO-HPVs, and LR-HPsV, HPV-52, HPV-58, HPV-16, and HPV-31 and HPV-68 being the predominant HR-HPVs, HPV-53 and HPV-66 the predominant PO-HPVs, and HPV-6 the predominant LR-HPV;
✓ The HR-HPV genotypes in FSWs matched generally with the HPV types targeted by the prophylactic 9-valent Gardasil-9^®^ HPV vaccine, reinforcing the widely use of this prophylactic vaccine for primary prevention of HPV infection and cervical cancer in the FSW key population;
✓ HIV and HSV-2 infections were associated with higher prevalences of genital shedding of any HR-HPV as well as multiple HR-HPVs, and with higher HPV viral load for several genotypes;
✓ By multiple linear regression analysis, the only strong predictor for significantly higher HR-HPV viral load, including Gardasil-9^®^ and non-vaccine HR-HPV, was genital co-infection by HSV-2;
✓ Prophylactic 9-valent Gardasil-9^®^ HPV vaccine in association with diagnosis and treatment of genital HSV-2 co-infection could be useful to prevent HPV-associated cervical lesions.

## 4. Discussion

We herein evaluated genital HPV shedding and co-factors among FSWs in Kisangani, DRC, a region with high burdens of HIV, HPV, and HSV-2. We also evaluated, for the first time in this population with a low education level, the acceptability and performance of the V-Veil UP2™ device for genital self-sampling. The veil collector demonstrated high acceptability (96%) and optimal effectiveness (100.0%), with all genital specimens yielding adequate cellular DNA (TOP3 gene positivity [55]) for HPV molecular testing. Five key findings emerged from this study. First, HIV prevalence (9.1%) was over 8-fold higher than national average, confirming FSWs as a key high-risk group for HIV and STIs. Second, HPV genotypes’ distribution appeared atypical and unique, with a mixture of HR-HPVs, PO-HPVs, and LR-HPVs, while HPV-52, HPV-58, HPV-16, and HPV-31 and HPV-68 were the predominant HR-HPVs. Third, 89% of HR-HPV-positive FSWs carried genotypes covered by the Gardasil-9^®^ vaccine, supporting the relevance of its use in that population. Fourth, co-infection with HIV and HSV-2 was linked with higher prevalence and viral loads of multiple HR-HPV genotypes. Finally, multivariate analysis identified genital HSV-2 infection as the sole independent predictor of increased HR-HPV replication. Together, these findings highlight the high HR-HPV burden in FSWs in the DRC, likely driven by genital HSV-2 shedding. Integrating HSV-2 detection and potential treatment could enhance cervical cancer prevention strategies in high-risk women living in Africa.

Given the high proportion of vaccine-preventable HPV types detected, strengthening Gardasil-9^®^ vaccination programs among FSWs is essential. Additionally, our findings support the integration of HSV-2 prevention, diagnosis, and treatment into cervical cancer screening and STI care, especially in high-risk populations. HSV-2 serology could serve as a triage tool to identify women at higher risk for persistent HR-HPV shedding and cervical disease. In the broader context of cervical cancer elimination efforts in sub-Saharan Africa, these results call for expanded, integrated prevention strategies, including HPV and HSV-2 screening, catch-up HPV vaccination, antiretroviral therapy for HIV, and self-sampling approaches to reach marginalized groups. Understanding the syndemic interaction of these infections is critical for reducing the burden of cervical cancer and improving outcomes among FSWs and other vulnerable populations across the region.

This study confirms the high HIV burden among FSWs in Kisangani, with seroprevalence of 9.1%, around 8-fold higher than the general adult female population in the DRC. This aligns with recent UNAIDS estimates among FSWs living in the DRC and in the West and Central Africa sub-region (7.5%) [7]. FSWs in the DRC, especially from Tshopo province, are a key core group for HIV infection. In Central and West Africa, HIV remains disproportionately concentrated among key populations, despite overall declines in incidence in the general population [3,6,8]. While the RDS method facilitated access to this hard-to-reach group [42,56], limitations related to network recruitment may affect representativeness [42,56] and thereby suggest the need for larger studies using alternative and less biased sampling strategies. Nevertheless, our findings suggest a substantial decline in HIV prevalence among Congolese FSWs compared to past estimates (26.3% in 2013) [3], though further monitoring is needed to determine if this trend is yet stabilized or sustained. Despite progress, access to HIV prevention and care remains limited in the DRC. Currently there is no effective so-called “minimum package of services” at the Congolese national level offering HIV testing, PrEP, and medical care tailored for FSWs in the DRC, although the target of 65% of FSWs reached by HIV prevention services was promoted by the National Multisectoral Program for the Fight Against HIV/AIDS [57]. These findings underscore the urgent need for expanded FSW-tailored interventions, to address HIV prevention and treatment gaps across the DRC and to reach the country’s zero incidence target by 2030 [7].

Geographical distribution of HPV genotypes provides specific molecular epidemiology of circulating HPV strains that vary widely across regions [58,59]. Accordingly, HPV genotyping among FSWs in Kisangani revealed a unique and atypical molecular epidemiology. Overall HPV prevalence (47.5%) in our study aligns with the global estimates among FSWs, as reported by Soohoo et al. (42.7%) [14] and Farahmand et al. (42.6%) [15], and reflects the high burden (20.0% to 29.6%) of genital HPV across African populations [60,61,62,63]. HR-HPV prevalence in our study reached 36.9%, closely mirroring rates reported among sub-Saharan African women attending health facilities for various gynecological problems (34.0%) [59].

Notably, the predominant relevant genotypes were HR-HPV-52 (16.6%) and HR-HPV-58 (11.1%), along with PO-HPV-53 (14.7%) and PO-HPV-66 (11.6%). HR-HPV-16 (6.7%), HR-HPV-18 (3.1%), and HR-HPV-45 (4.8%) were less common, suggesting a distinct atypical regional profile. These findings relatively differ from previous reports on African women highlighting HPV-16 (4.4%), HPV-52 (3.2%), HPV-35 (3.0%), and HPV-18 (2.8%) as the main four relevant genotypes [62]. Moreover, these observations are also relatively divergent from previous reports in the DRC and highlight significant intracountry variability. For instance, HPV-16 (33.3%) was dominant among HIV-positive women with CIN in Kinshasa [64], while HPV-68 prevailed in other Kinshasa cohorts [38,65,66]. In South Kivu, HPV-31 (43.7%), HPV-39 (27.6%), and HPV-16 (26.4%) were the most frequent genotypes [67]. Nevertheless, HPV-52 being the most common HR-HPV among FSWs in our study aligns with findings from Kinshasa [38,68]. HPV-58, rarely reported in Congo, ranked second, suggesting regional circulation in eastern DRC. HPV-16 was third, with inconsistent prevalence across studies [38,65,68]. HPV-18 and HPV-45 were generally infrequent in both our data and prior Congolese reports [38,65,68]. These findings highlight the relevance of Gardasil-9^®^ vaccine coverage in the region, as it includes the most prevalent HR-HPV types circulating among FSWs in eastern DRC. The high prevalence of HPV-53 (14.7%) in our study reinforces its potential importance in the region [38,65,68]. Although generally considered to have low oncogenicity, its frequent co-detection with other genotypes raises concern about potent synergistic effects [69]. Finally, the most prevalent LR-HPV was HPV-6, consistent with global trends among FSWs [14,15] and African women [38,61,62].

Importantly, one-third of participants had multiple HPV infections (two to eight HPV types), with 16.9% carrying multiple HR-HPV genotypes. These rates are considerably higher than those observed in women with normal cytology (3.2%) [61] or even invasive cervical cancer cases (13.2%) [61,70]. Multiple infections were also more frequent in HIV-positive women, consistent with previous research [71,72], and with findings from Senegalese FSWs, where the prevalence of multiple HPV infections reached 70.1% [73]. Multiple HPV infections are clinically significant. They are associated with increased risk of cervical abnormalities [74,75], poorer outcomes in invasive cervical cancer [76,77], and persistent low-grade lesions with potential progression to high-grade diseases [78,79]. Like what was previously reported in Nigeria [80], women with multiple infections in our cohort were more frequently HSV-2-positive, which may reflect immunomodulatory interactions between HPV and HSV-2, such as HPV-induced local immune suppression favoring HSV-2 replication [81,82]. HSV-2 co-infection may therefore exacerbate the risk profile for cervical disease progression. Moreover, HR-HPV infections are not only a cancer risk factor but also increase susceptibility to HIV acquisition [70,83,84]. HPV-infected, HIV-negative women are estimated to be twice as likely to acquire HIV [85], and FSWs with multiple HR-HPV infections face a 4-fold increased risk [85,86]. Thus, the high prevalence of multiple HR-HPV infections in this population may contribute both to cervical cancer risk and to ongoing HIV transmission dynamics. Taken together, these data underscore the urgency of comprehensive HPV prevention strategies, including expanded vaccination, regular screening, and integration with HIV and HSV-2 care for FSWs in the DRC.

Our findings also point out important public health implications for HPV prevention in Congolese FSWs. Although 60.5% of all detected HPV genotypes were not covered by the Gardasil-9^®^ vaccine, a substantial 90% of HR-HPV infections occurred with genotypes covered by the Gardasil-9^®^ vaccine, particularly HPV-52, HPV-58, and HPV-16. Furthermore, only 13% of HR-HPV-positive FSWs shed non-vaccine HR-HPV genotypes, indicating strong potential impact of Gardasil-9^®^ for cervical cancer prevention in this population. These data support shifting national HPV vaccination strategies in the DRC from the quadrivalent to the larger spectrum 9-valent vaccine, particularly for key populations like FSWs. However, vaccine-based prevention alone is insufficient, particularly for adult FSWs given the high prevalence of HR-HPV in this population, and the fact that existing vaccines may not fully match regional HPV strain distribution. A future goal in the improvement of cervical cancer prevention in sub-Saharan Africa should include the development of regionally adapted vaccines, particularly for women over 25 or those living with HIV. Simultaneously, scaling up molecular HPV screening and expanding existing pathology services for cytology and histology, as per updated World Health Organization (WHO) guidelines [87], remains critical but challenging in sub-Saharan African settings [88,89]. In that context, self-sampling offers a promising solution. The high acceptability (96%) and optimal effectiveness (100%) of veil-based genital self-collection observed in this study reinforces its potential for increasing coverage in hard-to-reach, marginalized populations [90,91,92,93,94]. Our findings build on our prior works from Chad and Spain, confirming that veil-based self-sampling is feasible, well accepted (96%), and suitable for molecular testing, with strong sensitivity (95.0%) and specificity (88.2%) [48,95]. Implementing such strategies, alongside vaccination and risk-reduction education, could greatly strengthen cervical cancer prevention efforts in FSWs. National programs must prioritize these integrated approaches to address structural barriers to care and ensure that vulnerable populations like FSWs are not left behind in the efforts toward cervical cancer elimination [96].

HR-HPV infection is the essential precursor for cervical cancer, yet only a fraction of infected women develop disease, suggesting that co-factors influence HPV persistence, viral replication, and carcinogenesis [97]. In this study we also examined both socio-demographic and biological predictors of HR-HPV shedding among FSWs in Kisangani, including HIV status and genital HSV-2 DNA detection, with quantitative viral load data providing insights into viral dynamics. Notably, we found that while HIV co-infection was associated with increased HR-HPV prevalence and viral burden, HSV-2 shedding emerged as the only independent predictor of elevated HR-HPV replication.

FSWs living with HIV showed higher prevalence of multiple HR-HPV genotypes, particularly HPV-31, HPV-33, HPV-39, HPV-56, HPV-52, and HPV-59, as well as significantly elevated viral loads of these genotypes and higher cumulative viral load across all HPV categories, including HPVs, HR-HPVs, PO-HPVs, Gardasil-9^®^ HR-HPVs, and non-vaccine HR-HPVs. These results align with previous findings in HIV-positive women across sub-Saharan Africa [32,98], where HIV-induced immunosuppression compromises the host capacity to clear HPV infection and facilitates viral persistence [98,99,100,101,102,103,104]. Mechanistically, HIV disrupts local and systemic immunity, impairs HPV-specific T cell responses, and alters mucosal integrity [24,105,106]. HIV also facilitates HPV replication at multiple stages, including virus entry, genome amplification, and evasion from immune surveillance [107]. Our results are consistent with large-scale studies across Africa that show HIV as a major driver of HPV infection, persistence, and associated disease. Indeed, studies from Morocco [108], Kenya [31,109], Cameroon [110], Senegal [111], Tanzania [112], and South Africa [113] all report significantly higher odds of HR-HPV detection and cervical dysplasia in HIV-infected women. In addition, HIV-positive FSWs in our study had elevated cumulative HPV viral loads, a potential biomarker of persistent infection and a known risk factor for CIN and cancer [114,115]. Higher HPV viral load, especially α-9 HPV-16 [116] and α-7 HPV-18 [117], is consistently associated with greater lesion severity in African studies, including Gabon [118], Burkina Faso [119,120], Kenya [31,121], Senegal [122], and South Africa [123,124].

However, despite the known synergy between HIV and HPV, multiple linear regression analysis revealed genital HSV-2 shedding, not HIV, as the sole independent predictor of elevated HR-HPV viral load. HSV-2-positive FSWs exhibited significantly higher prevalence of HR-HPV genotypes, especially HPV-16, -31, -45, -52, -58, and -59, and higher cumulative viral loads across all HPV categories. These associations have not previously been well described in African FSWs, making these findings novel and potentially impactful. HSV-2 was detected in 24.3% of FSWs overall and was 3.3 times more frequent among HIV-positive women. These rates correspond with known HSV-2 seroepidemiology in sub-Saharan Africa, the region with the highest global burden [125]. Seroprevalence among African FSWs reaches 65% [21,126], and women are biologically more susceptible to HSV-2 than men due to mucosal vulnerability and genital inflammation [127,128,129]. In our study, the asymptomatic nature of HSV-2 shedding was notable, as 20.1% of HIV-negative and 65.8% of HIV-positive women carried HSV-2 without symptoms, supporting the literature from Uganda, Ghana, and Central African Republic that shows frequent silent genital shedding in seropositive women [28,130,131,132]. This subclinical reactivation can persist for days and increases both HIV infectivity and transmission potential [22,23,133,134].

Biologically, HSV-2 may enhance HPV acquisition and persistence through mucosal disruption, chronic inflammation, and reduced epithelial barrier integrity [24,26,135]. These conditions create an ideal environment for HPV to access basal epithelial cells, facilitating initial infection and replication. Our finding of higher viral loads of oncogenic HPV genotypes in HSV-2-positive women supports this mechanism. Interestingly, HSV-2 also independently predicted elevated viral loads for Gardasil-9^®^-covered HR-HPV genotypes, reinforcing its clinical relevance for HPV control and cervical cancer prevention in FSWs.

Epidemiological studies corroborate this biological synergy. In China, HSV-2 cervical shedding was significantly more common among HPV-positive women (adjusted OR: 11.03) [34], while U.S. National Health and Nutrition Examination Survey data (2003–2010) linked HSV-2 seropositivity to increased HPV-16/18 prevalence [25]. A meta-analysis also reported that HSV-2 co-infection elevates risk of HPV-related cervical cancer and precancerous lesions [36,80]. Possible mechanisms include HSV-2-induced genomic instability, immunosuppression, and co-triggered inflammation that enhances HPV oncogenicity [26,136,137].

In our study, HPV genotypes with the highest viral loads in HSV-2-infected women included several with known carcinogenic potential, such as HPV-16, -31, -45, and -58, which are frequently detected in cervical cancer among African women [138]. These observations are critical because persistent high viral loads are associated with increased risk of CIN 2/3 and invasive carcinoma. In addition, co-infection with both HSV-2 and HIV appeared to amplify HPV replication even further. Our results support the hypothesis that HSV-2 may be a key driver of genital HR-HPV shedding, acting independently or synergistically with HIV [37,139].

Moreover, our data suggest that HSV-2-positive women are more likely to shed multiple HPV genotypes, a risk factor for persistent infection, cervical dysplasia, and poor treatment response [75,76]. HSV-2 may also alter the vaginal microbiome, promoting dysbiosis, immune dysfunction, and HPV persistence [26]. Taken together, these findings stress the importance of including HSV-2 testing and management in cervical cancer prevention programs for FSWs. In addition, the association of HSV-2 with higher replication of vaccine HPV genotypes reinforces the urgency of HPV vaccination for FSWs. Gardasil-9^®^ covers several of the most common HR-HPV types amplified in HSV-2-positive participants (HPV-16, -31, -45, -52, -58), suggesting that vaccination could meaningfully reduce the burden of HPV-associated diseases. Moreover, co-infections with HSV-2 and HIV may influence not only acquisition but also persistence and progression of HR-HPV infections, highlighting the need for integrated interventions. Therefore, we provide here strong evidence that HSV-2 is a potent and independent driver of HR-HPV replication in FSWs, possibly contributing to higher cervical cancer risk, even beyond the effects of HIV. Future research should examine whether HSV-2 suppression reduces HPV shedding and lesion progression and explore its inclusion in comprehensive cervical cancer prevention strategies tailored to high-risk populations like FSWs.

This study has several limitations. First, the representativeness of the included study population is not ensured. Indeed, the recruitment of participants using the RDS method by a single NGO in Kisangani focused on the care of key populations may have introduced several biases, including recruitment bias in which initial seeds may not be representative of the target population, referral bias in which participants may preferentially refer others similar to themselves, leading to oversampling of certain subpopulations, and network effects involving the social network structure which can influence recruitment patterns, potentially distorting the sample. Furthermore, the sample size of our study population was relatively small accentuating the risk of selection bias. Thus, the study participants may not be completely representative of the FSW community across the DRC, especially regarding the prevalence of HIV and genital HPV infection, as well as the distribution of HPV genotypes. The lack of a predetermined sample size may have also introduced recruitment bias that could affect the generalizability of study’s results. Since this is a cross-sectional study, the cause-and-effect relationships, especially between HSV-2 and HPV replication, cannot be definitely established. Additionally, voluntary participation may have introduced self-selection bias, while sensitive questions administered via face-to-face interviews may have affected response accuracy due to social desirability bias. Importantly, we did not assess cervical pathology, such as cytological or histological abnormalities, which limits the clinical significance of HPV viral load findings. Other unmeasured confounders, such as intestinal helminth co-infections common in sub-Saharan Africa, may also influence local immune responses and viral replication [140,141]. Lastly, HSV-2 and HPV share similar behavioral risk factors, raising the possibility of residual confounding despite our focus on FSWs, a population with relatively homogeneous sexual behavior patterns [142]. While these limitations constrain broad extrapolation, they do not undermine the key findings on HPV genotype distribution, HSV-2 as a predictor of HPV replication, and the feasibility of self-sampling [41].

## 5. Conclusions

This study highlights the high burden of HR-HPV infection among Congolese FSWs, characterized by an atypical genotype distribution and frequent co-infections with HIV and HSV-2. While both viruses were associated with increased HR-HPV prevalence and viral load, HSV-2 emerged as the sole independent predictor of higher HR-HPV replication, including both vaccine and non-vaccine genotypes. These findings reveal HSV-2 as a hidden epidemic that may not only facilitate HIV transmission and disease progression [30], but also act as a key cofactor in HPV persistence and pathogenesis. This genital syndemic interaction (HPV, HSV-2, and HIV) operates synergistically, reinforcing each other’s replication and increasing the risk of cervical precancerous lesions and cancer among FSWs.

## Figures and Tables

**Table 1:** Socio-demographic characteristics and sexual risk factors by HIV status in the 415 study adult female sex workers living in Kisangani, Democratic Republic of the Congo.

Variable	All FSWs (n = 415)	HIV-Negative (n = 377)	HIV-Positive (n = 38)	*p*-Value ^µ^
**Socio-demographics**				
**Age** (years)				
**All ages** [mean (SD)]	28.2 (8.1) [18–60] *	27.9 (7.8) [18–60]	30.7 (10.5) [19–60]	NS
**Ranges** [n (%)]				
18–24	152 (36.6)	139 (36.9)	13 (34.2)	NS
25–29	125 (30.1)	115 (30.5)	10 (26.3)	NS
30–39	98 (23.6)	91 (24.1)	7 (18.4)	NS
≥40	40 (9.7)	32 (8.5)	8 (21.1)	0.012
**Residency** [n (%)]				
Makiso	97 (23.4)	83 (22.0)	14 (36.8)	0.039
Tshopo	66 (15.9)	65 (17.2)	1 (2.6)	0.019
Mangobo	88 (21.2)	86 (22.8)	2 (5.3)	0.011
Kisangani	84 (20.2)	80 (21.2)	4 (10.5)	NS
Kabondo	80 (19.3)	63 (16.8)	17 (44.8)	<0.0001
**Religion** [n (%)]				
Christian	231 (55.7)	222 (58.9)	9 (23.7)	<0.0001
Muslim	61 (14.7)	52 (13.8)	9 (23.7)	NS
Kimbanguist	60 (14.4)	47 (12.5)	13 (34.2)	<0.0003
Other	63 (15.2)	56 (14.8)	7 (18.4)	NS
**Education level** [n (%)]				
Never attended school	82 (19.7)	73 (19.4)	9 (23.7)	NS
Primary school	172 (41.4)	150 (39.8)	22 (57.9)	0.031
Secondary school	129 (31.1)	123 (32.6)	6 (15.8)	0.032
University	32 (7.8)	31 (8.2)	1 (2.6)	NS
**Marital status** [n (%)]				
Unmarried	333 (80.2)	301 (79.8)	32 (84.2)	NS
Married	82 (19.8)	76 (20.2)	6 (15.8)	NS
**Occupation** [n (%)]				
Professional FSWs	137 (33.0)	119 (31.6)	18 (47.4)	0.048
Professional FSWs (bar waitress and hotel girl)	62 (14.9)	54 (14.3)	8 (21.1)	NS
Nonprofessional FSWs (occasional)	21 (5.1)	20 (5.3)	1 (2.6)	NS
Nonprofessional FSWs (regular)	195 (47.0)	184 (48.8)	11 (28.9)	0.019
**Income** [USD per day; n (%)]				
0–5	179 (43.1)	167 (44.3)	12 (31.6)	NS
6–10	138 (33.2)	132 (35.0)	6 (15.8)	0.020
>10	98 (23.7)	78 (20.7)	20 (52.6)	<0.0001
**Risk factors**				
**Number of sexual partner(s)** [in past 3 days; n (%)]				
1	223 (53.7)	210 (56.0)	13 (31.6)	0.011
[2–5]	146 (35.2)	128 (33.9)	18 (47.4)	NS
>5	46 (11.1)	38 (10.1)	8 (21.0)	0.040
**Recent unprotected sexual intercourse** [n (%)]	126 (30.4)	118 (31.3)	8 (21.0)	NS
**Previous HIV testing** [n (%)]	237 (57.1)	221 (58.6)	16 (42.1)	0.049
**Genital HSV-2 DNA positivity** [n (%)]	101 (24.3)	76 (20.1)	25 (65.8)	<0.0001

^µ^ Mann and Whitney U test, Pearson’s χ^2^ test, or Fisher’s exact test were used for comparisons between HIV-positive and HIV-negative FSWs. * The ranges are presented in brackets. Abbreviations: HIV: Human immunodeficiency virus; HSV-2: Herpes simplex virus type 2; NS: Not significant.

**Table 2:** Cervical HPV detection and genotyping by HIV and HSV-2 status in the 415 study adult female sex workers living in Kisangani, Democratic Republic of the Congo.

Variable	All FSWs (n = 415)	HIV-Negative (n = 377)	HIV-Positive (n = 38)	*p*-Value ^µ^	HSV-2-Negative (n = 314)	HSV-2-Positive (n = 101)	*p*-Value ^µ^
**Any HPV** [n (%)]	197 (47.5)	168 (44.6)	29 (76.3)	<0.0002	96 (30.6)	101 (100.0)	<0.0006
**Any LR-HPV** [n (%)]	66 (15.9)	58 (15.4)	8 (21.0)	NS	35 (11.1)	31 (30.7)	<0.0001
HPV-6	43 (10.4)	37 (9.8)	6 (15.8)	NS	17 (5.4)	26 (25.7)	<0.0001
HPV-11	10 (2.4)	10 (2.7)	0 (0.0)	NS	8 (2.5)	2 (1.9)	NS
HPV-81	13 (3.1)	11 (2.9)	2 (5.3)	NS	10 (3.2)	3 (2.9)	NS
**Any HR-HPV** [n (%)]	153 (36.9)	129 (34.2)	24 (63.1)	<0.0005	62 (19.7)	91 (90.1)	<0.0001
HPV-16	28 (6.7)	24 (6.4)	4 (10.5)	NS	9 (2.9)	19 (18.8)	<0.0001
HPV-18	13 (3.1)	13 (3.4)	0 (0.0)	NS	10 (3.2)	3 (2.9)	NS
HPV-31	25 (6.0)	16 (4.2)	9 (23.7)	<0.0002	6 (1.9)	19 (18.8)	<0.0001
HPV-33	12 (2.9)	8 (2.1)	4 (10.5)	0.017	7 (2.2)	5 (4.9)	NS
HPV-35	14 (3.4)	12 (3.2)	2 (5.3)	NS	10 (3.2)	4 (3.9)	NS
HPV-39	18 (4.4)	12 (3.2)	6 (15.8)	0.003	4 (1.3)	14 (13.9)	<0.0001
HPV-45	20 (4.8)	18 (4.8)	2 (5.3)	NS	11 (3.5)	9 (8.9)	0.034
HPV-51	9 (2.2)	7 (1.8)	2 (5.3)	NS	7 (2.2)	2 (1.9)	NS
HPV-52	69 (16.6)	58 (15.4)	11 (28.9)	0.040	20 (6.4)	49 (48.5)	<0.0001
HPV-56	10 (2.4)	7 (1.8)	3 (7.9)	0.020	6 (1.9)	4 (3.9)	NS
HPV-58	46 (11.1)	39 (10.3)	7 (18.4)	NS	22 (7.0)	24 (23.8)	<0.0001
HPV-59	12 (2.9)	8 (2.1)	4 (10.5)	0.017	2 (0.6)	10 (9.9)	<0.0001
HPV-68	13 (3.1)	11 (2.9)	2 (5.3)	NS	8 (2.5)	5 (4.9)	NS
**Any PO-HPV** [n (%)]	112 (26.9)	88 (23.3)	24 (63.2)	<0.0001	34 (10.8)	78 (77.2)	<0.0001
HPV-26	7 (1.7)	6 (1.6)	1 (2.6)	NS	5 (1.6)	2 (1.9)	NS
HPV-53	57 (14.7)	50 (13.3)	11 (28.9)	NS	15 (4.8)	42 (41.6)	<0.0001
HPV-66	48 (11.6)	35 (9.3)	13 (34.2)	<0.001	6 (1.9)	42 (41.6)	<0.0001
HPV-73	3 (0.7)	3 (7.9)	0 (0.0)	NS	2 (0.6)	1 (0.9)	NS
HPV-82	8 (1.9)	7 (1.8)	1 (2.6)	NS	7 (2.2)	1 (0.9)	NS
Multiple types of any HPV	130 (31.3)	107 (28.4)	23 (60.5)	<0.0005	46 (14.6)	85 (84.1)	<0.0001
Multiple types of HR-HPV	70 (16.9)	57 (15.1)	13 (34.2)	<0.003	26 (8.3)	44 (43.6)	<0.0001
Multiple types of PO-HPV	11 (2.7)	9 (2.4)	2 (5.3)	NS	1 (0.3)	10 (9.9)	<0.0001
**Vaccine targeted HPV** [n (%)]							
Any 4-valent vaccine types *	87 (20.9)	77 (20.4)	10 (26.3)	NS	41 (13.0)	46 (45.6)	<0.0001
Multiple 4-valent vaccine types	5 (1.2)	5 (1.3)	0 (0.0)	NS	2 (0.6)	3 (2.9)	NS
Any 9-valent vaccine types **	164 (39.5)	140 (37.1)	24 (63.2)	<0.002	73 (23.2)	91 (90.1)	<0.0001
Multiple 9-valent vaccine types	63 (15.2)	52 (13.8)	11 (28.9)	0.013	25 (7.9)	38 (37.6)	<0.0001

^µ^ Pearson’s χ^2^ test or Fisher’s exact test were used for comparisons between HIV-positive and HIV-negative FSWs, and between HSV-2-positive and HSV-2-negative FSWs. * The 4-valent Gardasil-4^®^ vaccine (Merck & Co. Inc., NJ, USA) is effective against HPV genotypes HPV-6, -11, -16, and -18. ** The 9-valent Gardasil-9^®^ vaccine (Merck & Co. Inc.) is effective against HPV genotypes HPV-6, -11, -16, -18, -31, -33, -45, -52, and -58. Abbreviations: HIV: Human immunodeficiency virus; HPV: Human papillomavirus; HR-HPV: High-risk HPV; LR-HPV: Low-risk HPV; HSV-2: Herpes simplex virus type 2; NS: Not significant; PO-HPV: Possibly oncogenic HPV.

**Table 3:** Cervical HPV quantification by HIV and HSV-2 status in the 415 study adult female sex workers living in Kisangani, Democratic Republic of the Congo.

Variable [mean ± SD log Copies/10^4^ Cells]	All FSWs (n = 415)	HIV-Negative (n = 377)	HIV-Positive (n = 38)	P ^µ^	HSV-2-Negative (n = 314)	HSV-2-Positive (n = 101)	P ^µ^
**LR-HPV load**							
HPV-6	0.58 ± 1.71	0.55 ± 1.68	0.86 ± 2.04	NS	0.35 ± 1.28	1.42 ± 2.31	<0.0001
HPV-11	0.14 ± 0.89	0.15 ± 0.93	0.00 ± 0.00	NA	0.15 ± 0.93	0.15 ± 0.93	NS
HPV-81	0.18 ± 1.00	0.16 ± 0.94	0.35 ± 1.49	NS	0.17 ± 0.97	0.19 ± 0.19	NS
**HR-HPV load**							
HPV-16	0.37 ± 1.41	0.35 ± 1.36	0.64 ± 1.89	NS	0.15 ± 0.91	1.07 ± 2.18	<0.0001
HPV-18	0.17 ± 0.94	0.18 ± 0.98	0.00 ± 0.00	NA	0.17 ± 0.94	0.15 ± 0.91	NS
HPV-31	0.31 ± 1.27	0.22 ± 1.04	1.31 ± 2.39	<0.0001	0.09 ± 0.69	1.00 ± 2.15	<0.0001
HPV-33	0.15 ± 0.87	0.11 ± 0.73	0.57 ± 1.69	<0.003	0.11 ± 0.75	0.26 ± 0.83	NS
HPV-35	0.18 ± 0.97	0.17 ± 0.94	0.30 ± 1.28	NS	0.17 ± 0.97	0.19 ± 1.47	NS
HPV-39	0.24 ± 1.16	0.18 ± 1.01	0.87 ± 2.07	<0.0002	0.06 ± 0.60	0.79 ± 1.93	<0.0001
HPV-45	0.27 ± 1.21	0.27 ± 1.22	0.28 ± 1.21	NS	0.19 ± 1.06	0.49 ± 1.14	0.028
HPV-51	0.12 ± 0.81	0.10 ± 0.74	0.31 ± 1.34	NS	0.12 ± 0.80	0.11 ± 0.97	NS
HPV-52	0.92 ± 2.08	0.84 ± 1.99	1.69 ± 2.70	0.023	0.33 ± 1.30	2.74 ± 2.75	<0.0001
HPV-56	0.13 ± 0.83	0.10 ± 0.72	0.45 ± 1.55	NS	0.10 ± 0.74	0.21 ± 1.26	NS
HPV-58	0.60 ± 1.72	0.56 ± 1.66	1.04 ± 2.24	NS	0.38 ± 1.39	1.29 ± 2.04	<0.0001
HPV-59	0.16 ± 0.95	0.12 ± 0.85	0.55 ± 1.64	<0.004	0.04 ± 0.49	0.54 ± 1.35	<0.0001
HPV-68	0.16 ± 0.94	0.16 ± 0.92	0.28 ± 1.20	NS	0.14 ± 0.85	0.27 ± 1.21	NS
**PO-HPV load**							
HPV-26	0.09 ± 0.71	0.08 ± 0.68	0.15 ± 0.95	NS	0.17 ± 0.94	0.09 ± 0.82	NS
HPV-53	0.81 ± 1.98	0.74 ± 1.90	1.58 ± 2.55	<0.0001	0.31 ± 1.27	2.37 ± 2.23	<0.0001
HPV-66	0.66 ± 1.77	0.54 ± 1.62	1.86 ± 2.64	<0.0001	0.09 ± 0.66	2.42 ± 2.74	<0.0001
HPV-73	0.04 ± 0.47	0.04 ± 0.49	0.00 ± 0.00	NA	0.04 ± 0.45	0.05 ± 0.84	NS
HPV-82	0.10 ± 0.75	0.09 ± 0.72	0.16 ± 0.99	NS	0.18 ± 0.79	0.06 ± 0.00	NS
**Cumulative HPV load**							
Any HPV	6.41 ± 9.18	5.72 ± 8.47	13.26 ± 12.69	<0.0001	3.36 ± 6.92	15.89 ± 8.07	<0.0001
LR-HPV	0.89 ± 2.08	0.86 ± 2.05	1.21 ± 2.41	NS	0.63 ± 1.79	1.72 ± 2.60	<0.0001
HR-HPV	3.81 ± 6.90	3.36 ± 6.25	8.28 ± 10.60	<0.0001	2.08 ± 5.70	9.16 ± 8.05	<0.0001
PO-HPV	1.71 ± 2.83	1.50 ± 2.71	3.77 ± 3.28	<0.0001	0.65 ± 1.81	5.00 ± 3.16	<0.0001
Gardasil-4^®^ HR-HPV ^$^	0.54 ± 1.81	0.53 ± 1.80	0.63 ± 1.89	NS	0.32 ± 1.44	1.23 ± 2.06	<0.0001
Gardasil-9^®^ HR-HPV ^£^	2.80 ± 4.88	2.53 ± 4.55	5.52 ± 6.82	<0.0003	1.44 ± 3.79	7.02 ± 5.68	<0.0001
Non-vaccine HR-HPV ^&^	1.00 ± 2.92	0.83 ± 2.66	2.75 ± 4.52	<0.0001	0.64 ± 2.52	2.14 ± 3.67	<0.0001

^µ^ Mann and Whitney U test was used for comparisons between HIV-positive and HIV-negative FSWs and between HSV-2-positive and HSV-2-negative FSWs. ^$^ The 4-valent Gardasil-4^®^ vaccine (Merck & Co. Inc., NJ, USA) is effective against HPV genotypes HPV-6, -11, -16, and -18. ^£^, The Gardasil-9^®^ vaccine HR-HPV genotypes were HPV-16, HPV-18, HPV-31, HPV-33, HPV-45, HPV-52, and HPV-58. ^&^ Non-vaccine HR-HPV genotypes were HPV-35, HPV-39, HPV-51, HPV-56, HPV-59, and HPV-68. Abbreviations: HIV: Human immunodeficiency virus; HPV: Human papillomavirus; HR-HPV: High-risk-HPV; LR-HPV: Low-risk HPV; HSV-2: Herpes simplex virus type 2; NA: Not attributable; NS: Not significant; PO-HPV: Possibly oncogenic HPV; SD: Standard deviation.

**Table 4:** Factors associated with cumulative viral loads of HR-HPV, Gardasil-9^®^-targeted HR-HPV, and non-vaccine HR-HPV in the 415 adult female sex workers living in Kisangani, Democratic Republic of the Congo, using multiple linear regression analysis.

	HR-HPV Cumulative Viral Load (log Copies/10,000 Cells)		Gardasil-9^® £^ HR-HPV Cumulative Viral Load (log Copies/10,000 Cells)		Non-Vaccine ^&^ HR-HPV Cumulative Viral Load (log Copies/10,000 Cells)	
	Coefficient β [95% CI]	*p*-Value ^$^	Coefficient β [95% CI]	*p*-Value	Coefficient β [95% CI]	*p*-Value
**Age**	0.017 [−0.06–0.10]	0.686	−0.009 [−0.06–0.05]	0.761	0.03 [−0.01–0.06]	0.179
**Residency**						
Kabondo	Ref.	–	Ref.	–	Ref.	–
Mangobo	1.47 [−2.45–1.81]	0.208	1.17 [−0.41–2.75]	0.145	0.29 [−0.75–1.34]	0.579
Kisangani	1.15 [−1.15–3.45]	0.326	0.41 [−1.18–1.99]	0.614	0.74 [−0.31–1.79]	0.164
Tshopo	1.89 [−0.43–4.22]	0.110	1.12 [−0.48–2.72]	0.172	0.78 [−0.28–1.84]	0.150
Makiso	1.91 [−0.12–3.93]	0.065	1.31 [−0.08–2.71]	0.066	0.59 [−0.33–1.52]	0.205
**Religion**						
Christian	Ref.	–	Ref.	–	Ref.	–
Muslim	−0.37 [−2.26–1.51]	0.697	−0.03 [−1.33–1.27]	0.961	−0.34 [−1.2–0.52]	0.435
Kimbanguist	0.76 [−1.19–2.71]	0.439	0.09 [−1.26–1.44]	0.894	0.67 [−0.22–1.56]	0.135
Other religion	0.32 [−1.62–2.26]	0.742	0.45 [−0.89–1.79]	0.504	−0.13 [−1.02–0.76]	0.771
**Education level**						
Never schooled	Ref.	–	Ref.	–	Ref.	–
Primary	0.68 [−1.12–2.47]	0.458	0.68 [−0.56–1.92]	0.276	−0.01 [−0.83–0.81]	0.984
Secondary	−0.04 [−1.97–1.9]	0.968	−0.12 [−1.46–1.21]	0.856	0.08 [−0.8–0.97]	0.852
University	−0.6 [−3.4–2.19]	0.67	0.06 [−1.87–1.99]	0.953	−0.66 [−1.93–0.61]	0.307
**Marital status**						
Married	Ref.	–	Ref.	–	Ref.	–
Unmarried	1.39 [−0.31–3.09]	0.107	0.72 [−0.46–1.89]	0.227	0.67 [−0.1–1.45]	0.088
**Occupation**						
Occasional non-FSW	Ref.	–	Ref.	–	Ref.	–
Regular non-FSW	0.14 [−3.27–3.54]	0.936	−0.008 [−2.36–2.34]	0.994	0.14 [−1.41–1.70]	0.852
Professional FSW	1.54 [−2.75–5.84]	0.481	0.57 [−2.39–3.54]	0.705	0.97 [−0.99–2.93]	0.331
Professional FSW in bars	−2.89 [−6.35–0.56]	0.101	−1.46 [−3.85–0.92]	0.229	−1.43 [−3.01–0.15]	0.076
**Daily income in dollars (USD)**						
[0–5]	Ref.	–	Ref.	–	Ref.	–
[6–10]	−0.02 [−1.77–1.74]	0.983	−0.002 [−1.21–1.21]	0.997	−0.02 [−0.82–0.78]	0.967
>10	1.98 [−0.93–4.89]	0.18	0.75 [−1.26–2.76]	0.464	1.23 [−0.09–2.56]	0.067
**Number of sexual partners in past 3 days**						
1	Ref.	–	Ref.	–	Ref.	–
[2–5]	−0.17 [−1.66–1.33]	0.825	−0.24 [−1.27–0.8]	0.653	0.07 [−0.61–0.75]	0.845
>5	−2.45 [−9.87–4.97]	0.514	−0.56 [−5.68–4.57]	0.83	−1.89 [−5.28–1.49]	0.269
**Recent unprotected** **sexual intercourse**	1.71 [−0.39–3.8]	0.109	1.3 [−0.15–2.75]	0.078	0.41 [−0.55–1.37]	0.398
**Previous HIV test**	−0.58 [−1.86–0.7]	0.37	−0.17 [−1.06–0.71]	0.698	−0.41 [−0.99–0.18]	0.168
**Genital HSV-2 positivity**	5.32 [3.54–7.1]	<0.001	4.59 [3.37–5.82]	<0.001	0.84 [0.12–1.55]	0.021
**HIV status**	2.01 [−0.47–4.48]	0.11	0.91 [−0.8–2.62]	0.295	1.1 [−0.03–2.23]	0.055

^$^ *p*-values were calculated using Pearson χ^2^ or Fisher exact tests. ^£^ Gardasil-9^®^ vaccine HR-HPV genotypes were HPV-16, HPV-18, HPV-31, HPV-33, HPV-45, HPV-52, and HPV-58. ^&^ Non-vaccine HR-HPV genotypes were HPV-35, HPV-39, HPV-51, HPV-56, HPV-59, and HPV-68. Abbreviations: CI: Confidence interval; HIV: Human immunodeficiency virus; HR-HPV: High-risk HPV; HSV-2: Herpes simplex virus type 2; Ref.: Reference category.

## Data Availability

The raw data supporting the conclusions of this article will be made available by the authors on request.
